# Volatile Constituents of Endophytic Fungi Isolated from *Aquilaria sinensis* with Descriptions of Two New Species of *Nemania*

**DOI:** 10.3390/life11040363

**Published:** 2021-04-19

**Authors:** Saowaluck Tibpromma, Lu Zhang, Samantha C. Karunarathna, Tian-Ye Du, Chayanard Phukhamsakda, Munikishore Rachakunta, Nakarin Suwannarach, Jianchu Xu, Peter E. Mortimer, Yue-Hu Wang

**Affiliations:** 1 CAS Key Laboratory for Plant Diversity and Biogeography of East Asia, Kunming Institute of Botany, Chinese Academy of Sciences, Kunming 650201, China; saowaluckfai@gmail.com (S.T.); samanthakarunarathna@gmail.com (S.C.K.); fungitianyed@163.com (T.-Y.D.); 2 World Agroforestry Centre, East and Central Asia, Kunming 650201, China; 3 Centre for Mountain Futures, Kunming Institute of Botany, Kunming 650201, China; 4 Yunnan Key Laboratory for Fungal Diversity and Green Development, Kunming Institute of Botany, Chinese Academy of Sciences, Kunming 650201, China; lu12364@outlook.com; 5 Institute of Plant Protection, College of Agriculture, Jilin Agricultural University, Changchun 130118, China; Chayanard91@gmail.com; 6 Engineering Research Center of Chinese Ministry of Education for Edible and Medicinal Fungi, Jilin Agricultural University, Changchun 130118, China; 7 State Key Laboratory of Phytochemistry and Plant Resources in West China, Kunming Institute of Botany, Chinese Academy of Sciences, Kunming 650201, China; munikishore@mail.kib.ac.cn; 8 Department of Biology, Faculty of Science, Chiang Mai University, Chiang Mai 50200, Thailand; suwan.462@gmail.com; 9 Research Center of Microbial Diversity and Sustainable Utilization, Faculty of Science, Chiang Mai University, Chiang Mai 50200, Thailand

**Keywords:** agarwood, chemical constituents, endophytic fungi, GC-MS analysis

## Abstract

Algae, bacteria, and fungi, as well as higher plants, produce a wide variety of secondary metabolites known as natural products. Natural products are well known as remarkable sources of many therapeutic agents. The genus *Nemania* is a wood-decaying fungus that belongs to family Xylariaceae. *Nemania* is often found as an endophyte in diverse hosts and some species are known to produce useful secondary metabolites. In this study, two *Nemania* species were isolated as an endophytic fungus from *Aquilaria sinensis*. Multi-gene phylogenetic studies showed that the newly described strains of *Nemania* are new to science, and this is the first report of *Nemania* from the host *Aquilaria*. One of the fermented species, *Nemania aquilariae* (KUMCC 20-0268), resulted in five sesquiterpenoids, which were previously reported from agarwood, and their structures were identified by gas chromatography-mass spectrometry (GC-MS). In addition, five different media were investigated in vitro to optimize conditions for growing the fungal biomass of *Nemania aquilariae* and *N. yunnanensis*.

## 1. Introduction

The genus *Aquilaria* Lam., belonging to the family Thymelaeaceae, consist of 31 accepted species according to the International Union for Conservation of Nature (IUCN) red list of threatened species [1], and 19 of them are recognized as agarwood-producing species [2,3,4,5,6,7,8]. *Aquilaria subintegra* Ding Hou, *A. malaccensis* Lam., *A. crassna* Pierre ex Lecomte, and *A. sinensis* (Lour.) Spreng. are major species capable of producing agarwood, which contains economically important essential oils [9]. At present, two native *Aquilaria* species, viz., *A. sinensis* and *A. yunnanensis* S. C. Huang, have been widely cultivated in Southeast Asia, while *A. sinensis* is primarily planted in southern China. The resinous heartwood of *A. sinensis* is well known for its medicinal importance in traditional Chinese medicine (TCM), named ChenXiang [10,11,12,13].

Heartwood contains resin-impregnated fragrant wood that is extremely valuable and in high demand throughout the world [14,15]. Healthy *Aquilaria* trees can only produce agarwood after being subjected to damaging events [3,5,16,17,18]. In natural forests, agarwood formation occurs slowly and infrequently in old trees, and only 7–10% of *Aquilaria* trees contain agarwood. When compared with market demand, the supply of agarwood from wild sources is severely inadequate. Unfortunately, indiscriminate felling of trees and overharvesting in hopes of finding the treasured resin have led to the severe depletion of wild trees and other negative impacts on biodiversity [19]. As a result, eight *Aquilaria* species are now listed on the IUCN red list as endangered species [1,20]. 

Many artificial induction approaches, viz., chisel nails, burning, trunk breaking, and bark removal, for the development of agarwood via traditional methods have been developed but these methods are slow and produce poor-quality agarwood [3,21,22]. Several techniques inducing agarwood production are described in Tan et al. [23]. So far, more than 300 compounds have been isolated and reported from agarwood and *Aquilaria* trees [24,25]. Fungal inoculum development in *Aquilaria* trees first began in 1929 [26]. Later, several researchers isolated fungi from naturally occurring *Aquilaria* trees in the wild (using healthy or diseased parts) to investigate the role of fungi in agarwood formation, finding that most of the isolated fungi were endophytes [22]. Fungi are some of the organisms involved in inducing agarwood formation, and fungal culture for inoculum can be “pure” or “mixed” [22]. For instance, *Fusarium laseritum*, *Lasiodiplodia theobromae,* and *Menanotus flavolives* are able to promote agarwood formation [25,26,27]. Outcomes may vary between fungal strains and across sites when they are applied.

**Figure 1:**
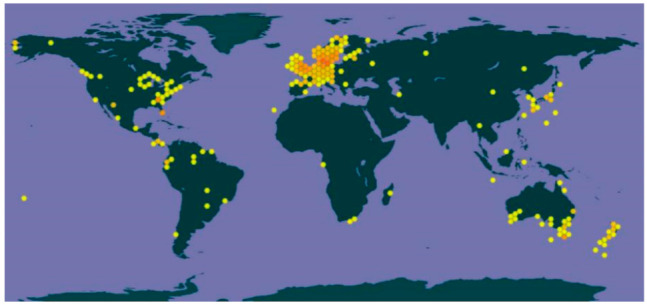
*Nemania* collection and distribution. High, moderate, and low *Nemania* samples’ collection is indicated in red to yellow gradient hexagons.

The genus *Nemania* is an endophytic fungal genus that has been reported on several hosts. Endophytic fungi of *Nemania* have shown interesting applications associated with its bioactive compounds on many hosts [28,29,30,31,32]. The pattern of *Nemania* geographic distribution from 1979–2020 is shown in Figure 1 [33]. This genus is distributed mostly in Europe (Denmark, Sweden, and the United Kingdom), Australia, and North America, while only few specimens have been recorded from Asia, Africa, and South America. This clearly shows that *Nemania* species are more diverse in temperate zones than tropical zones. 

The aim of this study was to isolate two endophytic fungi from the resin of *Aquilaria sinensis* collected from Yunnan Province, China. Multi-gene phylogenetic analyses showed two endophytic fungi are new species of *Nemania.* Besides, one of the species *N. aquilariae* fungally ferments was investigated for how its volatile organic compounds are formed, which is related to eventually forming agarwood, which was confirmed by GC-MS method. In addition, we optimized the best media for production of fungal biomass yields of the newly described strains’ isolates in vitro.

## 2. Materials and Methods

### 2.1. Sample Collection, Fungal Isolation, Preparation of Cultures, and Production of Fungal Biomass

Endophytic fungi species were isolated from dark resinous heartwoods of *Aquilaria sinensis* collected from Xishuangbanna Dai Autonomous Prefecture (N 21°44′38″, E 100°21′36″), Yunnan Province, China. Pieces of agarwood were burned to verify the presence of the agarwood fragrance before being stored in ice boxes and transported to the Kunming Institute Botany laboratory. Samples were cleaned under running tap water to remove dust and then air dried. Samples were cut into 0.5-cm, circular-shaped pieces. The surface of each sample was disinfected by being soaked in 75% ethanol for 1 min, 3% sodium hypochlorite solution for 2 min, and 75% ethanol for 30 s, followed by three rinses in sterile distilled water before finally being dried on sterile tissue papers [34]. All sections were placed in potato dextrose agar plates (PDA, Oxoid, Basingstoke, UK) and incubated at 28 °C for 1–3 days. Hyphal tips of fungal colonies appeared during incubation, so the colonies were transferred to new PDA plates and incubated to obtain pure cultures. New fungal taxa were examined in the pure culture, and photographs, morphological characteristics, and descriptions were completed.

For production of fungal biomass, fresh cultures of *Nemania aquilariae* and *N. yunnanensis* were inoculated into the following five liquid broth media (without agar): CzapekDox broth (CDB, oxoid), malt extract broth (MEB, oxoid), potato dextrose broth (PDB, oxoid), Richard broth (RB), and Sabouraud’s broth (SB). Broth media (100 mL) were prepared according to the manufacturer’s instructions, poured into clean, 150-mL flasks, covered with cotton lids and aluminum foil on the top, and sterilized via autoclaving at 121 °C for 30 minutes. The pure cultures (14 days old) on PDA were cut out near the margin by a 0.5-cm-diameter, sterilized cork borer. Five culture disks were transferred to each media flask (triplicate) under aseptic conditions. The flasks were inoculated at 28 °C on a rotary shaker at a speed of 120 rpm for seven days [35]. After seven days of incubation, mycelial masses were harvested via filtration through Grade 1 Whatman filter paper No. 1 (Madiston, Walton-on-Thames, UK) (initial weight of the filter papers was recorded prior to use), dried at 40–45 °C for 24 h, weighed of biomass, and recorded. Three replicates of biomass in various liquid media were carried out for each treatment, and data are the average of these three assays. Statistical analyses were performed using one-way ANOVA and the Mann–Whitney ranks sum test. Graphs and statistical analyses used Sigmaplot version 12.5 (Systat, San Jose, CA, USA). Analysis of variance of *p* ≤ 0.05 was used as the threshold for significance. Herbarium specimens were prepared from cultures that were dried in silica gel. The holotypes were deposited in Kunming Institute of Botany Academia Sinica (HKAS), Kunming, China. The ex-type cultures were deposited in the Kunming Institute of Botany culture collection (KMUCC). New taxa were registered in Facesoffungi (FoF) [36] and Index Fungorum [37]

### 2.2. Genomic DNA Extraction, PCR Amplification, and Sequencing

Genomic DNA was isolated from pure fungal cultures using the Biospin Fungus genomic DNA extraction kit-BSC14S1 (Bioflux, Kunming, China). Polymerase chain reaction (PCR) was used to amplify partial gene regions of Internal Transcribed Spacers (ITS), 28S ribosomal RNA (LSU), RNA polymerase II second largest subunit (RPB2), beta- tubulin (BT), and actin (ACT), using primers shown in Table 1. Total volume of PCR mixtures for amplifications was 25 µL [38]. Purification and sequencing of PCR products were performed by TsingKe Biotech, Kunming, Yunnan, China.

### 2.3. Phylogenetic Analyses

Sequence data generated in this study were checked for the quality of chromotagrams, and raw forward and reverse sequences were assembled using Geneious Pro.v4.8.5. Subjected to Basic Local Alignment Search Tool (BLAST) searches in the nucleotide database of GenBank (http://blast.ncbi.nlm.nih.gov, accessed on 16 April 2021) to determine their most probable closely related taxa. Sequence data were retrieved from GenBank based on BLAST searches and recent publications [44]. Sequence alignments were carried out through MAFFT v.6.864b [45] and the alignments were manually improved where necessary. Sequence data sets were combined using BioEdit [46].

**Figure 2:**
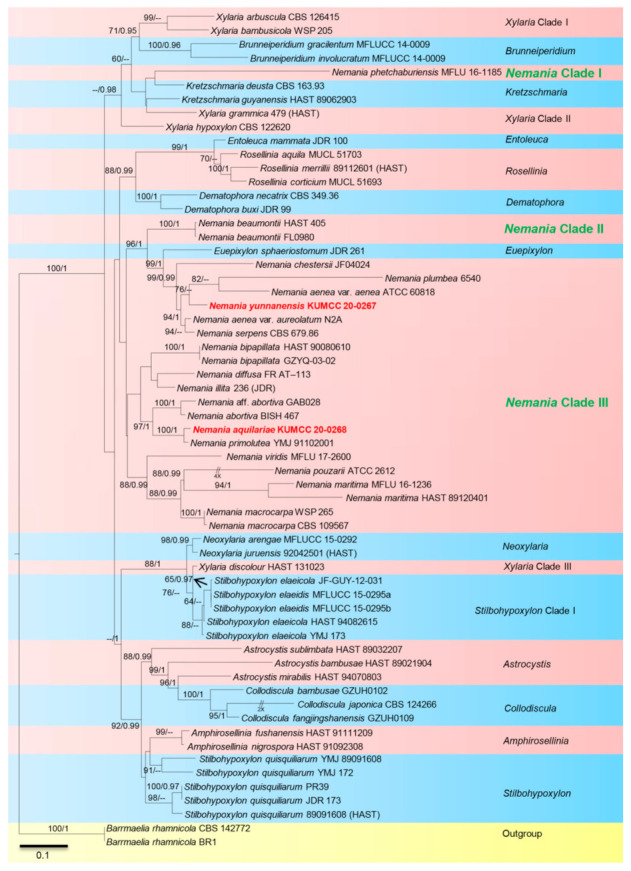
Phylogram generated from RAxML analysis based on combined ACT, ITS, LSU, BT, and RPB2 sequence data. Related sequences were obtained from Dayarathne et al. [44]. Bootstrap support values for ML equal to or greater than 60% and BYPP from MCMC analyses equal to or greater than 0.90 are given above/below the nodes. The ex-type strains are indicated in bold. Newly generated sequences are indicated in red bold.

Dissyanake et al. [47] were followed for construction of the combined phylogenetic trees using maximum likelihood (ML) and Bayesian Inference posterior probabilities (BYPP). The GTR+I+G model of nucleotide substitution and searches for model selected for ML were applied. Bootstrap supports were obtained by running 1000 pseudo-replicates. Bayesian Inference analysis was conducted when two parallel runs were performed using the default settings in addition to the following adjustment: Six Markov chains were run simultaneously for 5,000,000 generations, trees were sampled every 100th generation, and 20% of trees representing the burn-in phase were discarded. The remaining 80% of trees were used to calculate probability proportional to size (PPs). Bootstrap support values for ML and BYPP were given next to each node in the phylogenetic trees (Figure 2), which were configured in Fig Tree v1.4.0 [48] and edited using Microsoft Office PowerPoint 2019 and Adobe Photoshop CC 2019 (Adobe Systems, San Jose, CA, USA). Sequences of the new strains generated in this study were submitted to GenBank (Table 2).

### 2.4. Volatile Compound Analysis

Volatile organic compounds (VOCs) analysis was performed using a headspace solid-phase microextraction coupled with gas chromatography-mass spectrometry (HS-SPME-GC-MS). The GC-MS was equipped with a HS-SPME 50/30 µm divinylbenzene/carboxen/polydimethylsiloxane (PDMS/CAR/DVB) extraction head 57328-U (Sigma-Aldrich, St. Louis, MO, USA) connected with a headspace bottle (40 mL, 5190-4000, Agilent Technologies, Santa Clara, CA, USA). The GC-MS analysis was performed using the Agilent 5975C VL GC/MSD System with the 7890A GC System equipped with a DB-5ms capillary column (50 m × 0.25 mm, 0.25 μm, Agilent Technologies, Santa Clara, CA, USA).

### 2.5. Headspace Solid-Phase Microextraction (HS-SPME) Conditions

Fungal species determined in this study were recultivated on PDA and transferred to 40-mL headspace vials containing 5 mL of PDA solid medium (triplicate). VOCs were extracted by SPME, while uninoculated PDA headspace vial was set as the blank control. The inocula were incubated in a constant temperature incubator at 28 °C for 14 days for solid-phase microextraction. The silicon cap of the vial was closed and conditioned for 250 °C for 30 min by its insertion into the GC injection port under helium atmosphere. The extraction head was inserted into the headspace bottle and adsorbed for 30 min at room temperature (22 °C).

### 2.6. GC-MS Analysis Conditions for the Analyzation of VOC Emissions

The GC-MS conditions were set as follows: The initial temperature was set at 80 °C for 1 min and programmed to increase the temperature at a rate of 20 °C min^−1^ to 180 °C and then increase by 4 °C min^−1^ to 230 °C for 2 min. The inlet temperature was 250 °C, the connection port temperature was 290 °C, and desorption was performed for 5 min. Helium with a purity exceeding 99.999% was used as the carrier gas; the flow rate was set at 1.0 mL min^−1^ (spitless mode). The condition of mass spectrometry sources was set as connection temperature 280 °C. The ionization mode was electron ionization (EI), and the ionization temperature was 230 °C. The MS, four-stage rod temperature was 150 °C. Analyses were performed by setting the electron energy at 70 eV in full-scan mode (*m/z* 50–600). All identified components were quantified using NIST (National Institute of Standards and Technology) mass spectral database search and GC/MSD ChemStation data analysis software (Agilent Technologies, Santa Clara, CA, USA) and summarized as a percentage of relative peak area, shown in Table 3.

## 3. Results

### 3.1. Phylogenetic Analyses

The data set consisted of 58 strains included in the combined sequence analyses, comprising 5193 characters with gaps (349 bp ACT, 1938 bp BT, 841 bp ITS, 848 bp LSU, 1217 bp RPB2). *Barrmaelia rhamnicola* strains BR1 and CBS 142772 were used as outgroup taxa. Tree topology of the ML analysis was similar to the BYPP. The best scoring ML tree with a final likelihood value of –54284.745986 was presented. The matrix had 2725 distinct alignment patterns, with 50.86% undetermined characters or gaps. Estimated base frequencies were as follows: A = 0.234252, C = 0.278693, G = 0.245890, and T = 0.241164; substitution rates AC = 1.243781, AG = 4.000858, AT = 0.997311, CG = 1.026916, CT = 5.673379, and GT = 1.000000; and gamma distribution shape parameter α = 0.922891.

Phylogenetic analyses (Figure 2) showed newly described taxa group with *Nemania* species, and this genus separated into three clades: Clade I, *N. phetchaburiensis* (MFLU 16-1185), Clade II *N. beaumontii* (strains HAST 405 and FL0980), and most other *Nemania* species in clade III. Based on multi-gene phylogenetic analyses, newly described taxa grouped in clade III, *N. aquilariae* (KUMCC 20-0268) clustered with *N. primolutea* (MJ 91102001) with high support (100% in ML, 1.00 in BYPP) and *N. yunnanensis* (KUMCC 20-0267), were well separated from other species in *Nemania* with good support from ML (76% ML) but moderate support from BYPP (Figure 2).

### 3.2. Taxonomy

#### 3.2.1. *Nemania* Gray 1821

*Nemania* was erected for a heterogeneous assemblage of taxa by Gray [49] that belongs to family Xylariaceae. This genus is known to contain saprobes or endophytes from terrestrial or marine environments worldwide [50,51]. There are 55 records of *Nemania* in Species Fungorum [52]. 

#### 3.2.2. *Nemania aquilariae* Tibpromma & Lu, sp. nov.

**Figure 3:**
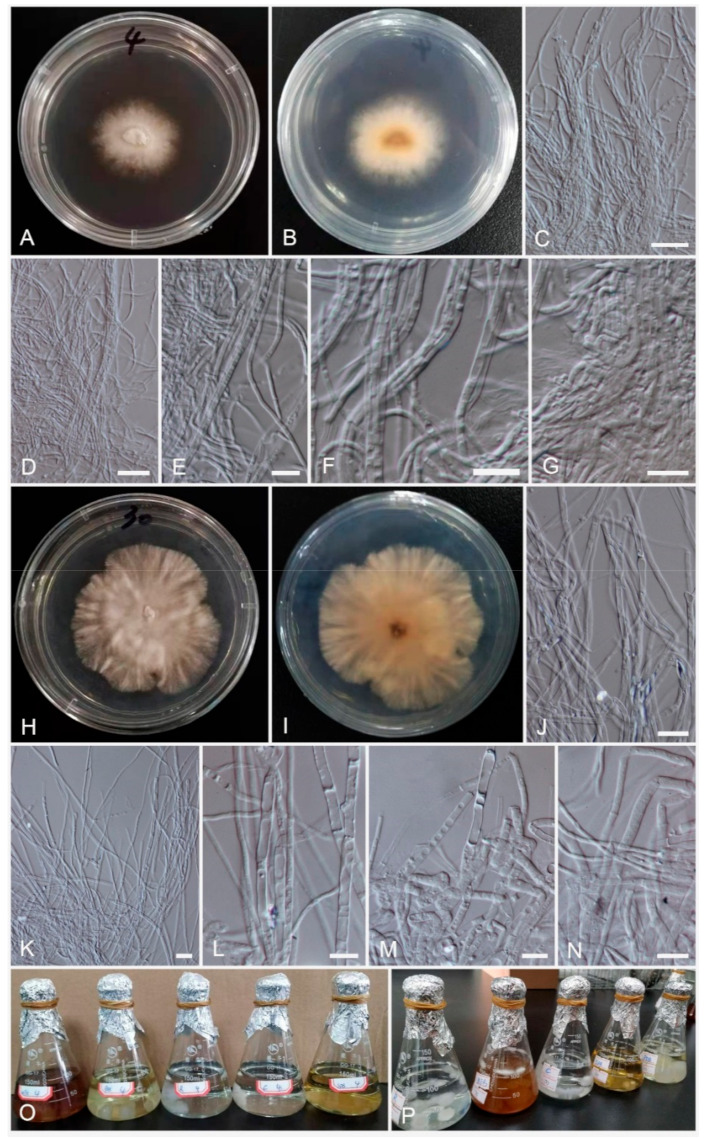
*Nemania yunnanensis* (KUMCC 20-0267, ex-type). (**A**,**B**) Colony on PDA at room temperature after seven days from above and below. (**C**–**G**) Mycelia masses. (**O**) Fermented in the various media. *Nemania aquilariae* (KUMCC 20-0268, ex-type). (**H**,**I**) Colony on PDA at room temperature after seven days from above and below. (**K**–**N**) Mycelia masses. (**P**) Fungal cultures growing in various media. Scale bars: (**C**,**D**,**J**,**K**) = 20 μm; (**E**–**G**,**L**–**N**) = 10 μm.

Index Fungorum number: IF558188; Facesoffungi number: FoF 09704; Figure 3H–N.

Etymology: name referring to the host genus *Aquilaria,* on which the fungus was found. 

Holotype: HKAS 111935

Culture characteristics: Colonies on PDA at room temperature (25 °C) reaching 9 cm in two week; circular, yellow-white with white margin; flossy, velvety, and raised; yellow-brown from below. Generative hyphae simple-septate, sub-hyaline, cells with guttules, thick-walled, 1.5–4 μm wide. Not sporulating in culture (Oatmeal agar (OMA) and PDA).

Material examined: CHINA, Yunnan Province, Xishuangbanna, on dark resinous wood of *Aquilaria sinensis* (Lour.) Gilg (Thymelaeaceae), 1 May 2019, Lu Z, No. 30 (HKAS 111935, holotype); ex-type living cultures, KUMCC 20-0268.

**Figure 4:**
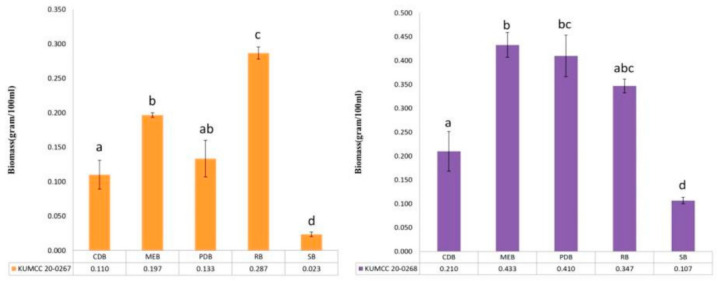
*Nemania yunnanensis* (KUMCC 20-0267, orange) and *N. aquilariae* (KUMCC 20-0268, purple) cultures fermented in different liquid media for seven days. All data are the averages of three measurements at 28 °C on a rotary shaker at 120 rpm. Letters indicate a significant difference (*p* ≤ 0.05, Mann–Whitney rank sum test) between different media. Error bars show standard error of the arithmetic mean.

Notes: Based on BLASTn searches of ACT, ITS, LSU, BT and RPB2 sequence data, *Nemania aquilariae* showed a high similarity to *N. primolutea* (ACT=98.62%(EF025592); ITS=99.65%(MG881830); BT=98.52%(EF025607), and RPB2=95.41%(GQ844767) while LSU showed high similarity to *N. beaumontii* 98.10% (MF161217). In the multi-gene phylogeny, *N. aquilariae* clustered sister to *N. primolutea* with 100% in ML and 1.00 in BYPP statistical support (Figure 2). The newly described strain is an endophytic fungus, which does not sporulate in culture so its morphological characteristics cannot be compared with *N. primolutea*. Sequence comparison results revealed 1.72% (ACT), 3.10% (RPB2), while other genes <1% base pair differences (without gaps) between *N. aquilariae* and *N. primolutea* (YMJ 91102001, holotype)*. Nemania primolutea* has also been found in China (Taiwan region) on dead trunk of *Artocarpus communis,* which differs from *N. chrysoconia* and *N. flavitextura* in having carbonaceous tissue between perithecia and absence of perithecial mounds [53]. Based on significant statistical supports in molecular phylogenetic studies, *N. aquilariae* is introduced herein as a new species on *Aquilaria sinensis* from Yunnan Province, China. In addition, MEB, PDB, and RB media are most ideal for culturing fungal biomass, while CDB and SB use led to the lowest level for culturing *N. quilariae* (Figure 4).

#### 3.2.3. *Nemania yunnanensis* Tibpromma & Lu, sp. nov.

Index Fungorum number: IF558189; Facesoffungi number: FoF 09705; Figure 3A–G.

Etymology: named after Yunnan Province, the place where the fungus was first discovered. 

Holotype: HKAS 111934

Culture characteristics: Colonies on PDA at room temperature (25 °C) reaching 9 cm in four weeks, circular, white, entire edge with raised on-media surface, smooth. Generative hyphae simple-septate, sub-hyaline, thin-walled, mycelium always packed together, 1.5–2 μm wide. Not sporulating in culture (OMA and PDA).

Material examined: CHINA, Yunnan Province, Xishuangbanna, on dark resinous heart wood of *Aquilaria sinensis* (Lour.) Gilg (Thymelaeaceae), 1 May 2019, Lu Z, No.4 (HKAS 111934, holotype); ex-type living cultures, KUMCC 20-0267.

Notes: *Nemania yunnanensis* well separates from other species in *Nemania* with moderate statistical support in ML analysis (Figure 2). Based on BLASTn searches of ACT, ITS, LSU, BT and RPB2 sequence data, *Nemania yunnanensis* showed a high similarity to *N. serpens* (ACT = 93.13%(KU684031); ITS = 98.70%(MN844431); BT = 90.84%(KU684188) and RPB2 = 92.60%(GQ844773)), while LSU sequence data showed high similarity to *N. beaumontii* 98.29%(MF161217). As newly described, the strain is an endophytic fungus and does not sporulate in culture. We were not able to compare morphological characteristics with other species in the genus. Based on phylogenetic analyses, *N. yunnanensis* is introduced herein as a new species on *Aquilaria sinensis* from Yunnan Province, China. In addition, the best media to support fungal biomass are RB and MEB media, while CDB, PDB, and SB media led to the lowest level for culturing *N. aquilariae* (Figure 4).

### 3.3. Screening Best Culture Media in Shake Flask Culture Method

Fresh and pure cultures of *Nemania yunnanensis* (KUMCC 20-0267) and *N. aquilariae* (KUMCC 20-0268) were used for fermentation in five different media. MEB, PDB, and RB media showed the highest dry weight for mycelium mass. For *Nemania yunnanensis* (KUMCC 20-0267), RB (38.22%) and MEB (26.22%) showed the highest mycelium mass followed by PDB (17.78%), CDB (14.67%), and SB (3.11%). For *Nemania aquilariae* (KUMCC 20-0268), MEB (28.76%), PDB (27.21%), and RB (23.01%) showed the highest mycelium mass followed by CDB (13.94%) and SB (7.08%) (Figure 4).

### 3.4. GC-MS Analyses

**Figure 5:**
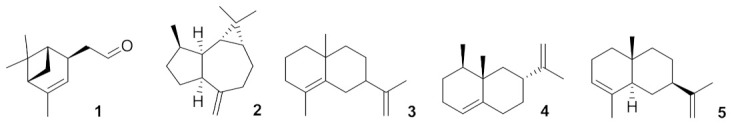
Chemical structures of volatile constituents of *Nemania aquilariae* (KUMCC 20-0268) detected by GC-MS. (**1**) Bicyclo[3.1.1]hept-3-ene-2-acetaldehyde, 4,6,6-trimethyl-, (1R,2R,5S) rel-. (**2**) Alloaromadendrene. (**3**) Naphthalene, 1,2,3,4,4a,5,6,7-octahydro-4a,8-dimethyl-2-(1-methylethenyl)-. (**4**) Valencen. (**5**) α-Selinene.

**Figure 6:**
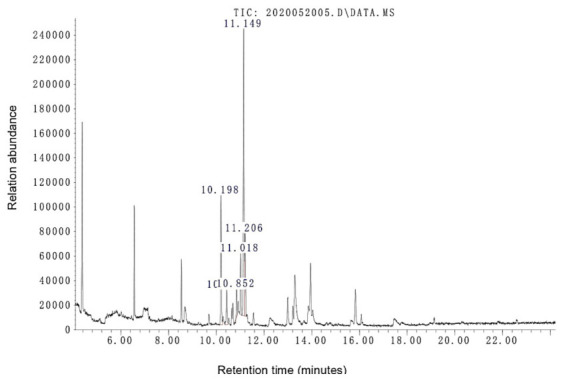
Typical gas chromatography–mass spectrometry (GC-MS) chromatogram (total iron current) of volatile constituents in fermented *Nemania aquilariae* (KUMCC 20-0268). The retention time (RT) refers to peak compounds and are listed in Table 3.

Five volatile components were found in *Nemania aquilariae*, accounting for 85.90% of total volatile components (Table 3 and Figure 4). All the five components’ structures were confirmed as sesquiterpenoids (Figure 5). However, no volatile components were detected in *Nemania yunnanensis* (data not shown). The dominant components of *Nemania aquilariae* were reported as compound 4 with 43.75%, compound 1 with 20.52%, and compound 5 with 13.38%, respectively (Figure 6).

## 4. Discussion

Several endophytic fungi are known as potentially bioactive metabolite producers in *Aquilaria* trees, and this is used in agarwood-producing trees [54,55,56]. In this study, two endophytic fungi belonging to *Nemania* were isolated from the dark resinous wood of *Aquilaria sinensis,* collected from Xishuangbanna, and this is the first report of *Nemania* from the host genus *Aquilaria.* Multi-gene phylogenetic analyses (Figure 2) showed that new isolates are new species of *Nemania.* It was also shown that *Nemania* species separated into three clades when more genes were included (Figure 2). So, we suggest protein-coding genes are important for resolving the placement of *Nemania*. In phylogenetic analyses (Figure 2), new isolates grouped in *Nemania* Clade III, and most of species in this clade were found on decorticated rotten wood and as endophytic fungi but they are not host specific [29,31,57]. Moreover, *Euepixylon sphaeriostomum* clusters within *Nemania* which is in consistent with Dayarathne et al. [44].

In this study, *N. aquilariae* was able to induce the formation of agarwood in *Aquilaria sinensis* and was capable of producing certain agarwood compounds, such as guaiane-type (2), eudesmane-type (3 and 5), and eremophilane-type (4), and these types of sesquiterpenoids are related to chemical constituents of agarwood [58]. Thus, *N. aquilariae* can be used in biological fermentation to produce agarwood-related compounds and can also be used to infect other *Aquilaria* plants in the production of agarwood. *Nemania aquilariae* is an endophytic fungus from *Aquilaria sinensis* and can be used as an alternative source for catalyzing the production of agarwood and its key natural ingredients. *Nemania aquilariae* was shown investigate the relationship between the chemistry and fungal associates of agarwood formed. The species presented that agarwood formation significantly affects the chemical and fungal constituents of agarwood in *A. sinensis*. In the present study, we indicated that *N. aquilariae* was able to produce the volatile compounds closely related to a primary determinant of agarwood properties. Thus, only few fungi are being tested for promoting agarwood formation. This species could further influence agarwood formation by injecting the fungi into the trunk, branches, or punch holes and then to subsequently inject fungi into the *Aquilaria* tree. Those techniques avoid severe damage to *Aquilaria* trees and also allow for easy agarwood collection. MEB, PDB, and RB nutrient broths are recommended for the cultivation of *N. aquilariae* for high yield and good quality of biomass.

## Figures and Tables

**Table 1:** Primer names, sequences, and references.

Gene	Primer	Primer Sequence	References
ITS	ITS5	5′-DDAAGTAAAAGTCGTAACAAGG-3′	[39]
	ITS4	5′-TCCTCCGCTTATTGATATGC-3′	
LSU	LROR	5′-ACCCGCTGAACTTAAGC-3′	[39]
	LR5	5′-TCCTGAGG-GAAACTTCG-3′	
RPB2	RPB2-5F	5′-GGGGWGAYCAGAAGAAGGC-3′	[40]
	RPB2-7cR	5′-CCCATRGCTTGYTTRCCCAT-3′	[41]
BT	T1	5′-AACATGCGTGAGATTGTAAGT-3′	[42]
	T22	5′-TCTGGATGTTGTTGGGAATC-3′	
ACT	512F	5′-ATGTGCAAGGCCGGTTTCGC-3′	[43]
	783R	5′-TACGAGTCCTTCTGGCCCAT-3′	

**Table 2:** Names, isolate numbers, and GenBank accession numbers of the fungal taxa used for the phylogenetic analyses of this study.

Species	Isolates	GenBank Accession Numbers				
		ITS	LSU	RPB2	BT	ACT
*Amphirosellinia fushanensis*	HAST 91111209	GU339496	N/A	GQ848339	GQ495950	GQ452360
*Amphirosellinia nigrospora*	HAST 91092308	GU322457	N/A	GQ848340	GQ495951	GQ452361
*Astrocystis bambusae*	HAST 89021904	GU322449	N/A	GQ844836	GQ495942	GQ449239
*Astrocystis mirabilis*	HAST 94070803	GU322448	N/A	GQ844835	GQ495941	GQ449238
*Astrocystis sublimbata*	HAST 89032207	GU322447	N/A	GQ844834	GQ495940	GQ449236
*Barrmaelia rhamnicola*	BR1	MF488991	MF488991	MF489000	MF489019	N/A
*Barrmaelia rhamnicola*	CBS 142772	MF488990	MF488990	MF488999	MF489018	N/A
*Brunneiperidium gracilentum*	MFLUCC 14-0011	KP297400	KP340542	KP340528	KP406611	N/A
*Brunneiperidium involucratum*	MFLUCC 14-0009	KP297399	KP340541	KP340527	KP406610	N/A
*Collodiscula bambusae*	GZU H0102	KP054279	KP054280	KP276675	KP276674	N/A
*Collodiscula fangjingshanensis*	GZU H0109	KR002590	KR002591	KR002592	KR002589	N/A
*Collodiscula japonica*	CBS 124266	N/A	MH874889	KY624273	KY624316	N/A
*Dematophora buxi*	JDR 99	GU300070	N/A	GQ844780	Q470228	N/A
*Dematophora necatrix*	CBS 349.36	MH855818	KF719204	KY624275	KY624310	N/A
*Entoleuca mammata*	JDR 100	GU300072	N/A	GQ844782	GQ470230	GQ398230
*Euepixylon sphaeriostomum*	JDR 261	GU292821	N/A	GQ844774	GQ470224	GQ389696
*Kretzschmaria deusta*	CBS 163.93	KC477237	KY610458	KY624227	KX271251	N/A
*Kretzschmaria guyanensis*	HAST 89062903	GU300079	N/A	GQ844792	GQ478214	GQ408901
*Nemania abortiva*	BISH 467	GU292816	N/A	GQ844768	GQ470219	GQ374123
*Nemania aenea var. aenea*	ATCC 60818	KC477240	N/A	N/A	N/A	N/A
*Nemania aenea var. aureolatum*	N2A	AJ390428	N/A	N/A	N/A	N/A
*Nemania aff. abortiva*	GAB028	KY250393	N/A	N/A	N/A	N/A
*Nemania aquilariae*	KUMCC 20-0268	MW729422	MW729420	MW717891	MW881142	MW717889
*Nemania beaumontii*	HAST 405	GU292819	N/A	GQ844772	GQ470222	GQ389694
*Nemania beaumontii*	FL0980	JQ760608	N/A	KU684243	KU684161	KU684065
*Nemania bipapillata*	HAST 90080610	GU292818	N/A	GQ844771	GQ470221	N/A
*Nemania bipapillata*	GZYQ-03-02	N/A	N/A	MK852275	MK852276	MK852274
*Nemania chestersii*	JF04024	N/A	DQ840072	DQ631949	DQ840089	N/A
*Nemania diffusa*	FR AT-113	DQ658238	DQ840073	DQ631947	DQ840088	N/A
*Nemania illita*	236 (JDR)	N/A	N/A	GQ844770	N/A	N/A
*Nemania macrocarpa*	WSP 265	N/A	N/A	GQ844776	GQ470226	GQ389698
*Nemania macrocarpa*	CBS 109567	MH862830	MH874423	N/A	N/A	N/A
*Nemania maritima*	HAST 89120401	N/A	N/A	GQ844775	GQ470225	GQ389697
*Nemania maritima*	MFLU 16-1236	MN047122	MN017886	N/A	N/A	N/A
*Nemania phetchaburiensis*	MFLU 16-1185	MN047124	MF615402	N/A	N/A	N/A
*Nemania plumbea*	6540	JQ846087	N/A	N/A	N/A	N/A
*Nemania pouzarii*	ATCC 2612	KC477228	N/A	N/A	N/A	N/A
*Nemania primolutea*	YMJ 91102001	N/A	N/A	GQ844767	EF025607	EF025592
*Nemania serpens*	CBS 679.86	KU683765	N/A	KU684284	KU684188	KU684088
*Nemania viridis*	MFLU 17-2600	MN047123	MN017887	N/A	N/A	N/A
*Nemania yunnanensis*	KUMCC 20-0267	MW729423	MW729421	MW717892	MW881141	MW717890
*Neoxylaria arengae*	MFLUCC 15-0292	MT496747	N/A	MT502418	N/A	N/A
*Neoxylaria juruensis*	92042501 (HAST)	GU322439	N/A	GQ844825	GQ495932	GQ438753
*Rosellinia aquila*	MUCL 51703	KY610392	KY610460	KY624285	KX271253	N/A
*Rosellinia corticium*	MUCL 51693	KY610393	KY610461	KY624229	KX271254	N/A
*Rosellinia merrillii*	89112601 (HAST)	GU300071	N/A	GQ844781	GQ470229	GQ398229
*Stilbohypoxylon elaeicola*	YMJ 173	EF026148	N/A	GQ844826	EF025616	EF025601
*Stilbohypoxylon elaeicola*	HAST 94082615	GU322440	N/A	GQ844827	GQ495933	GQ438754
*Stilbohypoxylon elaeicola*	JF-GUY-12-031	MF038896	N/A	N/A	N/A	N/A
*Stilbohypoxylon elaeidis*	MFLUCC 15-0295a	MT496745	MT496755	MT502416	MT502420	N/A
*Stilbohypoxylon elaeidis*	MFLUCC 15-0295b	MT496746	MT496756	MT502417	MT502421	N/A
*Stilbohypoxylon quisquiliarum*	YMJ 172	EF026119	N/A	GQ853020	EF025605	EF025590
*Stilbohypoxylon quisquiliarum*	89091608 (HAST)	EF026120	N/A	GQ853021	EF025606	EF025591
*Stilbohypoxylon quisquiliarum*	PR39	AY909023	N/A	N/A	N/A	N/A
*Stilbohypoxylon quisquiliarum*	JDR 173	EF026148	N/A	GQ844826	EF025616	N/A
*Stilbohypoxylon quisquiliarum*	YMJ 89091608	EF026120	N/A	GQ853021	EF025606	EF025591
*Xylaria arbuscula*	CBS 126415	KY610394	KY610463	KY624287	KX271257	N/A
*Xylaria bambusicola*	WSP 205	EF026123	N/A	GQ844802	AY951762	N/A
*Xylaria discolour*	HAST 131023	JQ087405	N/A	JQ087411	JQ087414	N/A
*Xylaria grammica*	479 (HAST)	GU300097	N/A	GQ844813	GQ487704	GQ427197
*Xylaria hypoxylon*	CBS 122620	KY610407	KY610495	KY624231	KX271279	N/A

**Table 3:** The volatile constituents ^a^ from fermented *Nemania aquilariae* (KUMCC 20-0268) by using GC-MS.

No.	Name of the Active Constituent	Retention Time (min)	Molecular Formula (MF)	Molecular Weight (MW)	Relative Peak Area(%) ± SD		Components Having Biological Properties (Based on CAS Data Only)
					Blank	KUMCC 20-0268	
1.	Bicyclo[3.1.1]hept-3-ene-2-acetaldehyde, 4,6,6-trimethyl-, (1R,2R,5S) rel-	10.198	C_12_H_18_O	178.27	-	20.52 ± 0.01	-
2.	Alloaromadendrene	10.436	C_15_H_24_	204.35	-	4.16 ± 0.01	Antibacterial and antimicrobial
3.	Naphthalene, 1,2,3,4,4a,5,6,7-octahydro-4a,8-dimethyl-2-(1-methylethenyl)-	10.852	C_15_H_24_	204.35	-	4.09 ± 0.01	-
4.	Valencen	11.149	C_15_H_24_	204.35	-	43.75 ± 0.05	-
5.	α-Selinene	11.206	C_15_H_24_	204.35	-	13.38 ± 0.01	Antibacterial
Peak area (%)					-	85.90 ± 0.04	

^a^: All the compounds have matching quality ≥80%, when compared with NIST mass spectral database.
